# Identification and validation of genes involved in gastric tumorigenesis

**DOI:** 10.1186/1475-2867-10-45

**Published:** 2010-11-24

**Authors:** Thangarajan Rajkumar, Neelakantan Vijayalakshmi, Gopisetty Gopal, Kesavan Sabitha, Sundersingh Shirley, Uthandaraman M Raja, Seshadri A Ramakrishnan

**Affiliations:** 1 Dept. of Molecular Oncology, Cancer Institute (WIA), 38, Sardar Patel Road, Chennai - 600036, India; 2 Dept. of Pathology, Cancer Institute (WIA), 38, Sardar Patel Road, Chennai - 600036, India; 3 Dept. of Surgical Oncology, Cancer Institute (WIA), 38, Sardar Patel Road, Chennai - 600036, India

## Abstract

**Background:**

Gastric cancer is one of the common cancers seen in south India. Unfortunately more than 90% are advanced by the time they report to a tertiary centre in the country. There is an urgent need to characterize these cancers and try to identify potential biomarkers and novel therapeutic targets.

**Materials and methods:**

We used 24 gastric cancers, 20 Paired normal (PN) and 5 apparently normal gastric tissues obtained from patients with non-gastric cancers (Apparently normal - AN) for the microarray study followed by validation of the significant genes (n = 63) by relative quantitation using Taqman Low Density Array Real Time PCR. We then used a custom made Quantibody protein array to validate the expression of 15 proteins in gastric tissues (4 AN, 9 PN and 9 gastric cancers). The same array format was used to study the plasma levels of these proteins in 58 patients with gastric cancers and 18 from patients with normal/non-malignant gastric conditions.

**Results:**

Seventeen genes (ASPN, CCL15/MIP-1δ, MMP3, SPON2, PRSS2, CCL3, TMEPAI/PMEPAI, SIX3, MFNG, SOSTDC1, SGNE1, SST, IGHA1, AKR1B10, FCGBP, ATP4B, NCAPH2) were shown to be differentially expressed between the tumours and the paired normal, for the first time. EpCAM (p = 0.0001), IL8 (p = 0.0003), CCL4/MIP-1β (p = 0.0026), CCL20/MIP-3α (p = 0.039) and TIMP1 (p = 0.0017) tissue protein levels were significantly different (Mann Whitney U test) between tumours versus AN & PN. In addition, median plasma levels of IL8, CXCL9/MIG, CCL3/MIP-1α, CCL20/MIP-3α, PDGFR-B and TIMP1 proteins were significantly different between the non-malignant group and the gastric cancer group. The post-surgical levels of EpCAM, IGFBP3, IL8, CXCL10/IP10, CXCL9/MIG, CCL3/MIP-1α, CCL20/MIP-3α, SPP1/OPN and PDGFR-B showed a uniform drop in all the samples studied.

**Conclusions:**

Our study has identified several genes differentially expressed in gastric cancers, some for the first time. Some of these have been confirmed at the protein level, as well. Some of these proteins will need to be evaluated further for their potential as diagnostic biomarkers in gastric cancers and some could be useful as follow-up markers in gastric cancer.

## Introduction

Gastric cancer is one of the common cancers seen in South India, ranked 2^nd ^among men and 5^th ^among women in the Chennai Metropolitan area [1]. Of the patients presenting to the tertiary Institution, more than 90% are advanced at presentation and only palliative management is feasible in these patients [2]. In 2005 and 2006, a total of 1239 gastric cancer patients were seen at the Institute, of which 211 patients had been previously treated elsewhere. Among the treatment naïve patients (n = 1028), 61% were locally advanced and 39% were with distant metastasis. In view of the advanced nature of the disease and due to poor performance status, only 91 patients came up for surgery with intent to cure. This highlights the problem of lack of early detection for the gastric cancer in India.

In Japan, which has a high incidence of gastric cancer, photofluorography screening is done as a screening program for the population, resulting in early detection of lesions, some confined to mucosa only [3]. Such a procedure is unlikely to be the solution in a large country such as India. In view of the subtle symptoms such as indigestion, gradual weight loss which are generally ignored, most patients present with advanced disease. It is therefore essential to develop reliable screening tests which can help in early detection of the disease. Serum based tests for Pepsinogen and H. pylori antibody have not gained widespread acceptance and have not been considered for screening individuals by the National Cancer Center, Tokyo, Japan [3]. The diagnostic test should be preferably blood or urine sample based and needs to be specific.

Among the major risk factors for gastric cancer, diet plays an important role. Consumption of salted food (fish and meat) and tobacco are associated with an increased risk [4-6]. Chronic atrophic gastritis and intestinal metaplasia have been considered as pre-malignant changes in the gastric mucosa. Tobacco smoking, H. pylori, diets with high content of salt, nitrites and nitrates, and low intake of fruits and vegetables are known risk factors for chronic atrophic gastritis [7]. The south Indian diet traditionally consists of high chilly content and deep fried food, with the oil used for frying undergoing several cycles of usage before being discarded and hence containing a high content of carcinogens [5,8,9].

Gastric cancers are predominantly adenocarcinomas and could be of three sub-types - Intestinal, Diffuse and Mixed [10]. The Intestinal type of gastric cancer is usually seen in the distal part of stomach, has precancerous stages and comprises of cohesive cancer cells forming gland like structures [11]. Most of the intestinal types are well to moderately differentiated (WHO Classification). The Diffuse type consists of individual cancer cells infiltrating and spreading far beyond its macroscopic borders. They are usually poorly differentiated to undifferentiated [12]. In Chennai, distal tumours are more common than proximal cancers.

Gene expression studies in different cancers have helped in identifying genes involved in the process of tumorigenesis [13]. Microarray studies comparing the gene expression differences between normal stomach and gastric cancer [14], between young and elderly gastric cancer patients' tumours [15], between primary tumour and metastatic lesions [16] have been reported. Our study compares the gene expression between apparently normal gastric tissues obtained from patients with non-gastric cancers (Apparently normal - AN), gastric tissue samples well away from the tumor and confirmed by frozen section not to have tumour cells (Paired normal - PN) and gastric cancers (Tumours - T). This is the first study to look into the gene expression patterns of gastric cancers in south Indian patients and validate some of these genes at the protein level for their potential as biomarkers for gastric cancer.

## Materials and methods

Five AN samples (2 from patients with Hypopharyngeal cancer, 1 from upper oesophageal squamous cell carcinoma, 1 from peri-ampullary carcinoma, 1 from pancreatic cancer) from patients who underwent stomach resection as part of their primary surgery were included in the study. In addition, 24 gastric cancers and 20 PN were included in the study. All the patients provided their informed consent for the study, which was approved by the Institutional Ethical committee.

The operative specimens were immediately processed and sections were taken for frozen section. Tumour samples with more than 70% cancer cells; PN and AN samples with no evidence of tumour cells and apparently normal morphology were included in the study.

In addition, blood samples were collected from individuals undergoing oesophagogastroduodenoscopy (OGDscopy) for dyspeptic symptoms and to rule out any upper gastrointestinal tract pathology. Of this, 58 were found to be gastric cancers, 6 were found to have benign pathology in the stomach (gastritis, benign ulcer) and 12 had a normal OGDscopy report. In 8 gastric cancer patients who underwent radical surgery, post-operative blood sample was also collected between day 7 and day 15 except two patients in whom the sample was collected at the time of their first follow-up after surgery (days 55 and 64).

### RNA extraction

The RNA was extracted from the tissue samples using the RNeasy RNA extraction kit (Qiagen, Gmbh, Hilden; Cat no: 74106) as per the manufacturer's instructions. The quality of the RNA used for micro-array analysis was checked using the Bioanalyzer and samples with a RNA Integrity Number (RIN) of 7 or more were included in the study. RNA was quantitated using NanoDrop™ ND1000 (NanoDrop Technologies, USA) spectrophotometer.

### Detection of H pylori

Analysis for H pylori in gastric tissue samples was performed using PCR as previously described [17]. PCR primers designed to amplify S2 region of Vac A gene in the H pylori genome were used for detection. The reaction amplifies an amplicon of approximately 194 bp length.

### Microarray experiment

1 μg of total RNA from the tumour/PN/AN sample and universal RNA (Stratagene; Cat no: 740000-41) were reverse transcribed using Array script at 42°C for 2 hrs to obtain cDNA using the Amino Allyl MessageAmp II aRNA amplification kit (Ambion, Austin, Tx; Cat no: AM1797). The cDNA was amplified, labelled, hybridized and slides scanned as described earlier [18].

**All the raw data files have been submitted to GEO with an assigned GEO accession number - GSE17154**.

### Microarray data analysis

The Foreground and Background Median intensity for Cy3 and Cy5, were imported into BRB-ArrayTools software [19] using the Import wizard function. Background correction was not done. Global normalization was used to median centre the log-ratios on each array in order to adjust for differences in labelling intensities of the Cy3 and Cy5 dyes. The data was analysed using the Class comparison module [20] in the BRB-ArrayTools software.

### Class Comparison in BRB-Array Tools

We identified genes that were differentially expressed among the 3 classes (tumour/PN/AN) using the Class Comparison module. Univariate F-test was used and the genes were considered statistically significant if their p value was < 0.001. In addition a two fold difference was required between the different classes.

### Quantitative Real time PCR

Real time validation of the gene expression was done using the TLDA real time PCR (Applied Biosystems, Foster City, CA; Cat no: 4342261). Triplicate cDNA template samples were amplified and analysed on the ABI Prism 7900HT sequence detection system (Applied Biosystems, Foster City, CA) as described earlier [18].

The raw data from the Prism 7900HT sequence detection system was imported into Microsoft Excel software for statistical analysis of the data. Among the endogenous reference genes included on the array (18S ribosomal gene; ACTB), ACTB was chosen after visualizing the global Ct value distribution, for normalizing the data. In addition, HSPE1 which had been included based on the differential expression seen on Microarray, was found to have minimal variation and hence was included as an additional endogenous control. The TLDA assays were run at LabIndia Instruments Pvt Ltd laboratories at Gurgaon, New Delhi.

The AN samples were used as calibrators and the relative quantitation values were calculated for all the genes and the samples.

### Gene Ontology Analysis

The genes found to be differentially expressed in tumours and paired normals' based on the microarray data were imported into the Fatigo module of Babelomics [21] and analysed for over-representation of the GO terms in comparison to the rest of the genome, using Fisher's exact test for 2 × 2 contingency tables. The p value was set at < 0.05.

### Preparation of Tissue protein lysates and Collection of Plasma

Protein lysates for analysis using antibody arrays were prepared from 60-80 mg of frozen gastric tissue samples. The tissue samples were ground in the presence of liquid nitrogen using mortar and pestle. The powdered tissue was re-suspended in tissue lysis buffer (Tris. HCL pH7.5, 150 mM Sodium chloride, 1% Sodium deoxycholate, 1%NP40). Prior to use, lysis buffer was supplemented with Complete mini™ protease inhibitor cocktail (Roche Diagnostics GMBH, Germany). The protein lysates were subjected to sonication using Vibra cell™ (Sonics Inc., USA) sonicator. The lysates were cleared by centrifugation and quantitated using Coomassie Plus-Bradford assay ™ reagent (Pierce Inc., USA) as per manufacturer's protocol. The quality of the proteins was analysed by resolving 50 μg of the lysate on 10% sodium dodecyl sulfate polyacrylamide (SDS) gel and subsequently visualized by staining using Coomassie Blue.

Plasma was obtained from 5 ml of blood collected in the presence of 200 μl of 10% ethylene diamine tetra acetic acid (EDTA). The plasma isolated from the blood samples was centrifuged at 3000 g and stored at -80°C in aliquots.

### Antibody Arrays

Custom antibody arrays Quantibody ™ array (Catalogue number: QAA-CUST) based on a multiplex ELISA system for quantitative measurement of multiple proteins purchased from Ray Biotech, Inc, USA was used to study protein expression levels in gastric tissues and plasma samples [22,23]. The following genes (CXCL5/ENA-78, CXCL8/IL8, CXCL10/IP10, CXCL9/MIG, CCL3/MIP-1α, CCL15/MIP-1δ, EpCAM, MMP3, SPP1/OPN, TIMP1, Adipsin/CFD, CCL4/MIP-1β, CCL20/MIP-3α, PDGFR-B and IGFBP-3) found to be overexpressed in gastric cancers relative to PN and AN (with the exception of CFD/Adipsin which was overexpressed in PN relative to gastric cancers and AN) were studied for their protein levels in tumour, corresponding PN and in AN tissues. In addition, plasma levels of these proteins in patients who had undergone OGDscopy were estimated using the same array format (58 gastric cancer patients, 6 with benign gastric or duodenal ulcer or gastritis and 12 with normal OGDscopy report). In 8 gastric cancer patients who had undergone radical surgery, pre and post-operative samples were collected and analyzed.

The assay was performed as described by the manufacturer's protocol. Briefly, in the case of the tissue lysates 100 μg of the protein lysate was prepared by diluting the tissue lysate stock solution using sample diluent buffer supplied by the manufacturer to a final concentration of 1 mg/ml. 100 μl of the diluted lysate was incubated in the array chamber for 2 hours. In the case of the plasma samples, the plasma was diluted with sample diluent buffer supplied with the kit at a ratio of 1:1. 100 μl of the diluted plasma sample was incubated in the array chamber for 2 hours. For every array experiment, standards supplied along with the kit were freshly prepared as described by the manufacturers protocol and included along with a sample diluent buffer control (negative control) which neither had standard or samples. The arrays were processed as described by the manufacturer's protocol. The slides were scanned at 5 μ resolution and a PMT of 70 using Pro Scan Array™ (Perkin Elmer Inc., USA). At these scanner settings the signals from the highest standard concentration did not reach saturation. The data was analyzed using the Quantibody Q-Analyzer, an array specific, Microsoft Excel based program, supplied with the custom arrays.

### Statistical analysis

The median and range for the plasma values were calculated using Microsoft Excel ™ spread sheet software (Microsoft, Inc.,). Mann Whitney U test (http://faculty.vassar.edu/lowry/utest.html) was used to study the significance of the median values of the plasma and two tailed test was used for obtaining the p value.

## Results

The clinico-pathological details of all the patients, whose samples were used for the microarray analysis and their tumour samples, are given in Additional File 1 and 2. The clinico-pathologic details of the patients from whom blood samples were obtained for estimation of the plasma levels of cytokines/chemokines/growth factors is given in Additional File 3.

Of the 24 patients whose tumour samples were used for microarray study, 16 were aged more than 45 years of age and 8 were 45 years or less; 18 were males and 6 were females; 5 were tumours arising in the region of cardia, 2 from body and 17 from antrum. All the twenty-four tumours were adenocarcinomas with one of them being poorly differentiated adenocarcinoma with areas of neuro-endocrine features. Among the adenocarcinomas, Intestinal subtype was the most common (n = 16), while diffuse subtype was seen in 5 and mixed in 2. Grade III tumours predominated (n = 20), with no Grade I tumours in our series. Seventeen of 24 were node positive and 4 had distant metastasis at presentation.

Based on the Class Comparison analysis (p value = 0.001 and 2 fold difference), we had 61 genes overexpressed in cancer, 66 in paired normal and 61 with a p value of <1e-07 in apparently normal (Additional File 4). We used Taqman Relative Quantitation RT-PCR for validation of some of the genes identified by the microarray analysis. ACTB was chosen as an endogenous control, which was in addition to the 18S control present in the TLDA card.

All the 49 samples worked in the TLDA assay but 2 genes, C11orf42 and IGLL1 had not worked. HSPE which had been found to have differential expression in the samples in our microarray analysis did not show any variation in the levels, in the TLDA assay and was included as an additional endogenous control. Normalization was done using ACTB and HSPE as endogenous controls. Of the 63 genes selected, excluding the endogenous controls (ACTB and HSPE) and the two genes which had not worked, we had 59 genes for further analysis.

Three of the genes (REG4, CLDN18, MXRA5) had been included for validation of their potential as prognostic marker for failure. However, in the interval between the time that the microarray analysis was completed and the RQ-RT-PCR analysis was done (approximately 3 months) there were additional patients who relapsed and hence the genes were not considered for further analysis as prognostic markers. However, they were included to assess whether they were differentially expressed between PN and tumours.

**Figure 1:**
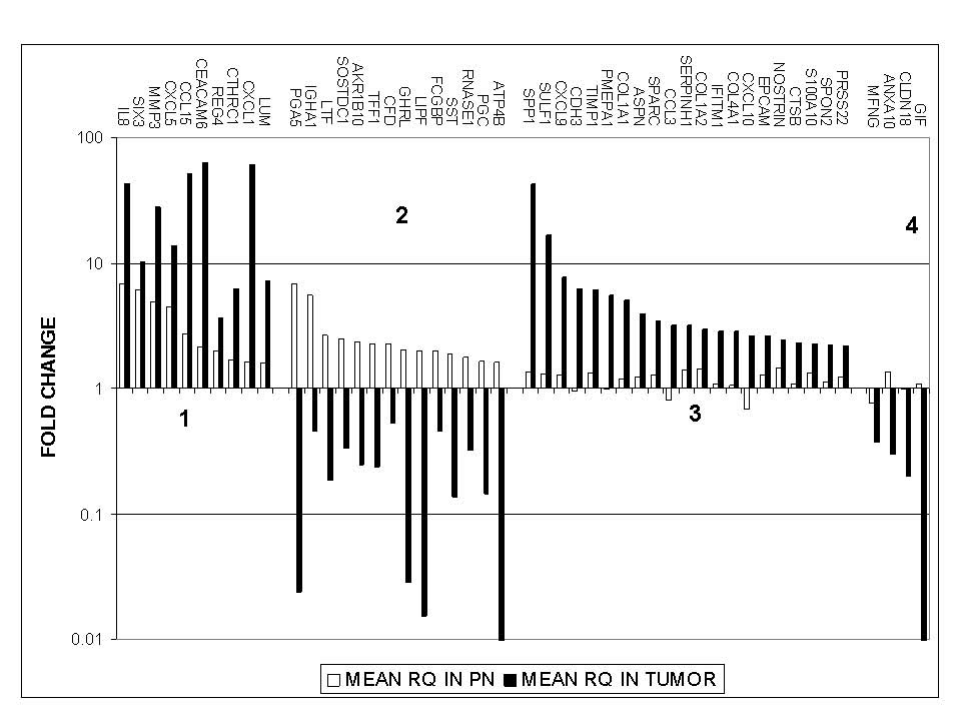
**RQ values of Paired normals (PN) (n = 20) and Tumour (n = 24), using apparently normal (AN) (n = 5) as calibrator**. The fold change is relative to the apparently normals.

The list of genes which were identified to be differentially expressed in gastric cancers are given in Table 1 and Table 2. Table 1 lists the genes identified to be differentially expressed in gastric cancer for the first time and Table 2, lists the genes which have been known to be associated with gastric cancer, and found also in our study. (Additional Files 5 and 6 provide information on these genes and the corresponding references). There are four different patterns of gene expression - Pattern 1 - overexpressed in PN and in Tumours; Pattern 2 - overexpressed in PN but downregulated in tumours; Pattern 3 - minimal change in PN but overexpressed in Tumours and Pattern 4 - minimal change in PN but downregulated in Tumours (Figure 1).

**Table 1:** Genes reported to be differentially expressed for the first time in gastric cancer [References and details in Additional File 4]

SNO	GENE SYMBOL	UPREGULATION REPORTED IN	REFERENCE
1	CCL15/MIP5/MIP-1d/LKN1	NSCLC	[SF5-R1]
2	ASPN	breast cancer	[SF5-R2]
3	MMP3	colorectal cancer	[SF5-R3]
4	SPON2	Lung, ovarian, prostate cancers	[SF5-R4] [SF5-R5] [SF5-R6]
5	PRSS22	ovarian cancer	[SF5-R7]
6	CCL3/MIP 1A	oral SCC	[SF5-R8]
7	TMEPAI/PMEPAI	Neuro-endocrine tumours	[SF5-R9]
8	SIX3		
9	MFNG	Downregulated in cervical cancer	[SF5-R10]
10	SGNE1/SCG5	endocrine tumors	
11	SOSTDC1	Downregulated in renal cancer	[SF5-R11]
12	SST	Downregulated in oesophageal adenocarcinoma	[SF5-R12]
13	IGHA1		
14	AKR1B10	Increased in Barrett's epithelium	[SF5-R13]
15	FCGBP	Down regulated in colon ca	[SF5-R14]
16	ATP4B		
17	NCAPH2		

**Table 2:** Genes known to be involved in gastric cancer tumorigenesis identified in this study [References and details in Additional File 5]

S NO	GENE SYMBOL	UP OR DOWN REGULATED	REFERENCE
1	CTSB	Up-regulated especially in cardia tumours	[SF6-R1]
2	SPARC/Osteonectin	Up-regulated	[SF6-R2]
3	COL1A1	Up-regulated	[SF5-R3]
4	COL1A2	Up-regulated	[SF5-R3]
5	COL4A1/Arresten	Up-regulated	[SF5-R4]
6	CXCL1/GRO1/MGSA	Up-regulated	[SF5-R5]
7	SPP1/osteopontin	Up-regulated	[SF5-R6]
8	CXCL9/MIG	Up-regulated	[SF5-R7]
9	IL8/CXCL8	Up-regulated	[SF5-R8]
10	TIMP1	Up-regulated	[SF5-R9]
11	LUM/SLRR2D/LDC	Up-regulated	[SF5-R10]
12	CXCL5/ENA78	Up-regulated	[SF5-R11]
13	CXCL10/INP10/IP10	Up-regulated	[SF5-R4]
14	CEACAM6	Up-regulated	[SF5-R12]
15	REGIV	Up-regulated	[SF5-R13]
16	S100A10/ANX2L/CAL1L	Up-regulated	[SF5-R14]
17	SERPINH1/HSP47/CBP1	Up-regulated	[SF5-R15]
18	CDH3	Up-regulated	[SF5-R16
19	TACSTD1/EPCAM/CD326	Up-regulated	[SF5-R17]
20	IFITM1/LEU13	Up-regulated	[SF5-R18]
21	CTHRC1	Up-regulated	[SF5-R19]
22	SULF1	Up-regulated	[SF5-R20]
23	RNASE1	Down-regulated	[SF5-R21]
24	PGC	Down-regulated	[SF5-R22]
25	PGA5	Down-regulated	[SF5-R22]
26	GIF	Down-regulated	[SF5-R23]
27	LTF	Down-regulated	[SF5-R24]
28	TFF1	Down-regulated	[SF5-R25]
29	CLDN18	Down-regulated	[SF5-R26]
30	CFD/Adipsin	Secreted by gastric ca cell lines	[SF5-R27]
31	GHRL/Obestatin	Down-regulated	[SF5-R28]
32	LIPF	Down-regulated	[SF5-R29]
33	ANXA10	Down-regulated	[SF5-R30]

We then proceeded to confirm the protein expression for some of the genes in the AN (n = 4), PN (n = 9) and tumours (n = 9). We used the Quantibody array, which is based on the principle of sandwich ELISA to determine the protein levels of 15 cytokines, chemokines and growth factors [22,23]. The Median values and the Range of the levels are given in Table 3. CFD/Adipsin median levels were found to be lower in tumours compared to the levels in PN and AN (p = 0.0324), while CXCL5/ENA78, CCL20/MIP-3α, IGFBP3, SPP1/OPN and TIMP1 were increased in PN and tumours relative to AN, with higher levels seen in tumours. In contrast, EpCAM, IL8, CXCL10/IP10, CCL3/MIP-1α, CCL4/MIP-1β, CCL15/MIP-1δ, PDGFR-B were predominantly elevated in tumours compared to AN and PN. EpCAM (p = 0.0001), IL8 (p = 0.0003), MIP-1β (p = 0.0026), MIP-3α (p = 0.039) and TIMP1 (p = 0.0017) levels were significantly different (Mann Whitney U test) between tumours versus AN & PN. Additionally, EpCAM (p = 0.0004), IL8 (p = 0.0015) and MIP-1β (p = 0.0061) were significantly elevated in tumours compared to their corresponding PN.

**Table 3:** Median values for cytokines and chemokines in an, pn and tumour lysates

	AN	AN	PN	PN	TUMOUR	TUMOUR
	Median in pg/ml	Range in pg/ml	Median in pg/ml	Range in pg/ml	Median in pg/ml	Range in pg/ml
**CFD/Adipsin**	27295.0	22097.9-31759.9	27162.9	19515.1 - 38224.3	13021.0*	9441.5 - 36873.1
**CXCL5/ENA-78**	283.8	39.3-4206.1	2922.7	84.9 - 31628.7	3978.0	723.9 - 40669.6
**EpCAM**	1466.0	905.7-2266.6	2243.5	915 - 8885.8	18717.6***/^**###**^	8966.1 - 34529
**IGFBP-3**	3149.8	124.0 - 17530.2	12286.0	134.6 - 34036.3	17662.7	196.9 - 55671.5
**IL-8/CXCL8**	22.1	0 - 62.3	53.1	27.4 - 190.6	1240.4***/^**###**^	93.1 - 3660.7
**CXCL10/IP-10**	20.4	1.4 - 146.1	147.0	0.8 - 3940.9	384.4	0.7 - 1643.1
**CXCL9/MIG**	369.5	0 - 8569.7	4336.1	1077.2 - 19396.6	7540.3	727.7 - 73975.9
**CCL3/MIP-1α**	0.0	0 - 187.4	203.6	0 - 1177.2	359.0	0 - 3231.5
**CCL4/MIP-1β**	49.5	0 - 261.8	94.3	0 - 335.8	578.6**/^**#**^	28.7 - 1185.2
**CCL15/MIP-1δ**	86.0	54 - 157.3	169.3	51.7 - 748.9	374.3	63.4 - 2825.4
**CCL20/MIP-3α**	859.3	76.1-1784.8	3049.6	893 - 16262.9	4773.4*	1014.4 - 13280.7
**MMP-3**	269.0	0 - 648.6	143.9	0 - 353.1	0.0	0 - 2908.2
**SPP1/OPN**	447.0	9.1 - 896.3	2054.0	193.6 - 3183.7	1407.1	12.6 - 5819.2
**PDGF R-B**	222.3	174.3 - 521.4	295.3	184.9 - 1079.3	578.6	123.7 - 1516.8
**TIMP-1**	9676.2	4373.3 - 17133.2	27022.2	17028.3 - 194387.8	139365.6**	48146.4 - 367203.2

AN - Apparently normal; PN - Paired normal

p value significant for AN+PN versus Tumour -

* - <0.05; ** - <0.005; *** - <0.0005 by Mann Whitney U test

p value significant for PN versus Tumour -

^# ^- <0.01; ^## ^- <0.005; ^### ^- <0.0005 by Mann Whitney U test

**Figure 2:**
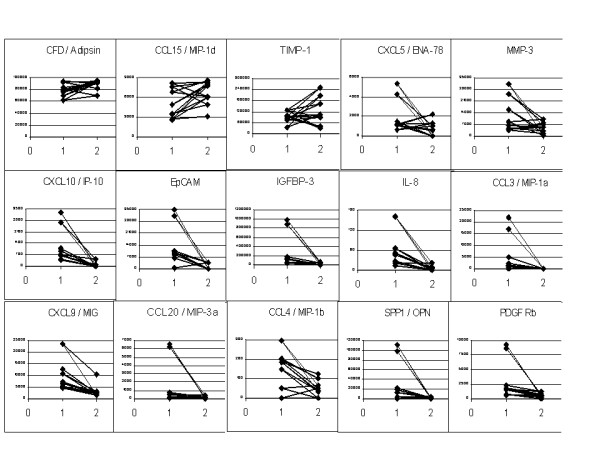
**Pre (1) and Post-operative (2) plasma levels of the cytokines/chemokines/growth factors in gastric cancers (n = 8)**. The plasma levels were measured using the Quantibody array, which uses the principle of sandwich ELISA in a multiplex format. The details for the estimation of the levels are provided in the Methods section.

The plasma levels of the 15 cytokines/chemokines/growth factors were estimated using the Quantibody array. The 18 non-malignant cases (12 with no abnormality detected by OGDscopy and 6 with benign pathology in the stomach) were clubbed together and the median values were compared between the tumours and the non-malignant group. Mann Whitney U test was performed to assess the statistical significance (two tailed test). IL8, CXCL9/MIG, CCL3/MIP-1α, CCL20/MIP-3α, PDGFR-B and TIMP1 plasma levels were significantly different between the non-malignant group and the gastric cancer group (Table 4). SPP1/OPN level were also higher in gastric cancer patients compared to the non-malignant group but was borderline significant (p = 0.05). The post-surgical levels of EpCAM, IGFBP3, IL8, CXCL10/IP10, CXCL9/MIG, CCL3/MIP-1α, CCL20/MIP-3α, SPP1/OPN and PDGFR-B showed a uniform drop in all the 8 samples studied. In contrast TIMP1, CCL15/MIP-1δ and CFD/Adipsin levels actually increased in the post-operative period in most of the samples (Figure 2).

**Table 4:** Median values for cytokines and chemokines in plasma from non-malignant patients versus plasma from patients with gastric carcinomas

	MEDIAN for non-malignant (n = 18) (in pg/ml)	RANGE (in pg/ml)	MEDIAN for tumours (n = 58) (in pg/ml)	RANGE (in pg/ml)
**CFD/Adipsin**	71520.0	58000.8 - 77527.9	69236.4	27926.9 - 95255.7
**CXCL5/ENA-78**	1100.6	0 - 7203.2	1702.8	0 - 11588.5
**EpCAM**	1789.8	0 - 13627.3	3527.1	0 - 35009.2
**IGFBP-3**	119467.1	20164.6 - 174498	110395.1	0 - 979359.6
**IL-8/CXCL8**	22.5	0 - 69.9	48.9*	0 - 396.3
**CXCL10/IP-10**	670.2	0 - 3328.9	931.1	0 - 5158.9
**CXCL9/MIG**	6069.2	0 - 18739.7	7561.8*	505-6 - 86999
**CCL3/MIP-1α**	1464.8	0 - 10602.2	2335.1*	0 - 32199
**CCL4/MIP-1β**	43.0	0 - 440.1	127.9	0 - 2138.5
**CCL15/MIP-1δ**	6628.8	1237.8 - 13178.1	5577.9	696.3 - 11054.4
**CCL20/MIP-3α**	139.9	0 - 1994.1	**654.6#**	0 - 12013.1
**MMP-3**	10966.2	3891.2 - 38928.9	13680.1	1358.7 - 80588.8
**SPP1/OPN**	4621.5	0 - 20518.1	8680.5	0 - 110373.2
**PDGF R-B**	1391.5	0 - 5461.8	2300.5*	0 - 34754.4
**TIMP-1**	31048.9	15603.8 - 220729.6	**98054.6#**	6252.5 - 463941

^**# **^- p value < 0.01; * - p value < 0.05 by Mann Whitney U test

The plasma levels of the cytokines/chemokines/growth factors were then correlated with the clinico-pathologic features. 28 of the 58 gastric cancer patients had undergone a potentially curative radical surgery (R0 resection) and 6 patients underwent R1 resection only. These 34 pathologic specimens were available for pathologic correlations (pT, pN, pStage, perinodal spread, lymphatic emboli, vascular emboli), while survival analysis was done on 28 patients who had undergone a R0 resection. Ten patients underwent palliative surgical procedures while 13 patients declined surgery or were unfit for any intervention other than supportive care. One patient had an undifferentiated cancer, which on additional Immunohistochemical studies was found to be a primary gastric lymphoma, diffuse large B cell type. Plasma levels for MMP3 where higher in males compared to females (Median value 16772.1 pg/ml versus 8512.5 pg/ml) (p value = 0.011); patients with tumours in the cardia were found to have higher plasma levels of MIG compared to other sites [antrum, pylorus and body] (Median value of 18203.9 pg/ml versus 7204.1 pg/ml) (p value = 0.032); TIMP1 levels were higher in tumours arising from the antrum and pylorus compared to those arising from the body and cardia of the stomach (Median value 125639.5 pg/ml versus 90820 pg/ml) (p value = 0.026); IP10, MMP3 and MIP-3α plasma levels were higher in patients with tumours which had lymphatic emboli compared to those which were negative (Median values of 1691.4, 17761.2, 2103.8 versus 543.8, 8359.2, 250.7 pg/ml, respectively) (p value 0.009, 0.009, 0.018, respectively). Plasma level of MMP3 was also associated with vascular emboli but this did not achieve statistical significance. The plasma levels of the cytokines were not significantly associated with disease-free or overall survival in the 28 patients who underwent radical curative surgery.

## Discussion

This is the first gene expression study on gastric cancer from south India, which has a high incidence rate for gastric cancers in India. As discussed earlier, diet related factors play an important role in the pathogenesis of these cancers [4-6,8,9].

In this study we have compared the gene expression profile of apparently normal (gastric tissue obtained from patients undergoing gastric resection for Non-gastric cancers), paired normal (gastric tissue well away from the gastric tumour and confirmed not to have any tumour cells) and the gastric tumours. Most studies have used the paired normal and the tumour for studying the gene expression patterns [14,24,25] while others have compared biopsies from erosive gastritis, atrophic gastritis, intestinal metaplasia and gastric cancers [26]. A few studies have used Laser capture micro-dissection to profile the gastric cancer cells [27].

Our study has identified for the first time several genes (n = 17) as being differentially expressed in gastric cancers (Table 1). In addition, 33 genes known to have been involved in gastric cancer were confirmed in our study as well (Table 2). Additional Files (5 and 6) indicate the known function of the genes obtained from Gene Cards [28].

Our study identified a large number of genes associated with the extracellular matrix (SPARC, SERPINH1/HSP47, COL1A1, COL1A2, COL4A1, MMP3, TIMP1, PRSS2, CDH3, SPP1, IL8, CTSB, LUM, CEACAM, CTHRC1, SULF1, ASPN, SPON2 and CLDN18) and chemokine activity, to be differentially expressed in gastric cancers.

SPARC, SERPINH1 and CTHRC1 are involved in collagen synthesis; while MMP3, CTSB are involved in breakdown of extracellular matrix, and TIMP could inhibit MMP activity. PRSS2 converts inactive MMP3 into active MMP3 while SPARC can activate MMP2. TIMP has additional tumour promoting role in oncogenesis, stimulating proliferation [29] and inhibiting apoptosis [30,31] thereby contributing to the malignant phenotype. ASPN, an extracellular matrix protein, which inhibits TGF-β and BMP2, is overexpressed in the gastric cancers relative to the paired normal [32].

REG4 has been reported to be a marker for peritoneal metastasis in gastric cancer [33] and this was supported by our study which showed that REG4 overexpression is associated with metastatic disease. SERPINH1 (HSP47) was also associated with metastatic disease and had been shown earlier to be a marker for metastatic tumour cells [34].

Some of the tumour suppressor genes identified in our study included Lactotransferrin (LTF), MFNG and SOSTDC1. LTF has been found to inhibit invasiveness of the gastrointestinal tumour cell lines [35]. MFNG has been shown to inhibit Jagged-Notch signalling in cervical cancer cells. Introduction of MFNG into the CaSki cervical cancer cell line leads to inhibition of its tumorigenicity [36].

Several cytokine mRNAs' (CCL3/MIP-1α, CCL15/MIP-1δ, CXCL1, CXCL5/ENA78, CXCL8/IL8, CXCL9/MIG, CXCL10/IP10) were found to be overexpressed in the tumours while CCL15/MIP-1δ, CXCL5/ENA78 and IL8 mRNAs' were overexpressed in paired normals, as well. However, at the protein level, there was up-regulation of CXCL5/ENA78, CCL20/MIP3-α, IGFBP3, OPN and TIMP1 in tumours and their PN, indicating that these could be early events in the gastric tumorigenesis. Park et al., (2007) [37] had reported CXCL5/ENA78 overexpression to be associated with late stage gastric cancer and nodal metastasis, using immunohistochemistry (IHC). Our study, using a more sensitive assay, suggests CXCL5/ENA78 to be involved in early phases of tumourigenesis as well. Interestingly, CXCL5/ENA78 was found to induce a proliferative response in non-transformed prostate cell lines [38]. CCL20/MIP-3α has been known to be expressed in inflamed gastric tissue and plays a role in H.pylori induced gastritis [39]. Using IHC, IGFBP3 was found to be overexpressed in gastric cancers relative to the normal gastric tissue [40]. SPP1/OPN has also been reported to be overexpressed in gastric cancers and increasing levels have been detected with progression of disease [41]. TIMP1 is an inhibitor of matrix metalloproteinases but at the same time can also induce cell proliferation and has an anti-apoptotic effect. It has been reported to be overexpressed in gastric cancer cells and in the inflammatory cells of the stromal element of the tumour [42].

EpCAM, IL8, CXCL10/IP10, CCL3/MIP-1α, CCL4/MIP-1β, CCL15/MIP-1δ, PDGFR-B protein were found to be expressed predominantly in tumours only, suggesting that they could be involved in the later stages of gastric tumorigenesis. EpCAM is expressed by most epithelial cell membranes and in gastrointestinal carcinomas. It has been shown to be overexpressed in gastric carcinomas and when downregulated by siRNA, it leads to decrease in cell proliferation, cell cycle arrest and suppressed tumour formation in nude mice [43]. IL8 is a potent pro-angiogenic and inflammatory chemokine [44] and has been found to play a role in the inflammatory response to H.pylori [45]. It has also been shown to be involved in the invasiveness and angiogenesis of several cancers including pancreatic cancer [46]. Of the three related Macrophage inflammatory proteins (CCL3/MIP - 1α, CCL4/MIP-1β and CCL15/MIP-1δ), CCL4/MIP-1β was the most differentially expressed. This beta-chemokine has been reported to be highly expressed in diffuse type gastric cancers [47]. Inspite of the production of these MIP proteins, the immune response to the tumours is not effective probably due to functional defects in the effector cells [48]. Chung et al (1992) [49] reported the presence of PDGFR-B mRNA and protein in the gastric tumours (using in situ hybridization and IHC) but not in the adjacent non-malignant mucosa, which is similar to our data.

We had chosen a large number of potentially secreted proteins for our validation of the microarray data using Taqman Real Time PCR (TLDA assay). Subsequently we evaluated 15 proteins which are secreted, for validation using the Quantibody protein array, both in gastric tissues and in the plasma. The Median values and the range of the 15 proteins in the plasma are given in Table 4. CCL20/MIP-3α, TIMP1, IL8, PDGFR-B, CCL3/MIP-1α and CXCL9/MIG were significantly elevated in the plasma from the gastric cancer patients compared to the plasma from individuals with no visible upper gastro-intestinal pathology or those with benign lesions in stomach. As in our study, TIMP1 levels have been found to be elevated in gastric cancer patients compared to healthy controls [50,51]. CXCL5 levels were found to be higher in late stage gastric cancer patients compared to levels in patients with benign conditions [37].

The pre- and post-operative paired plasma samples from gastric cancer patients who underwent radical surgery showed concordant drop for most of the proteins studied, except TIMP1, MIP-1δ and Adipsin. TIMP1 levels are known to be elevated following tissue injury. TIMP1 levels have been found to be elevated in the immediate post-operative period after curative resection for colo-rectal cancers and it took between 28 - 60 days to fall below the pre-operative levels [52]. Parsons et al., (2004) [53] have shown TIMP1 to be involved in decreased degradation of extracellular matrix (ECM) by blocking matrix metalloproteinase activity and enhanced survival of hepatic stellate cells which are a major source of ECM, leading to fibrosis in liver following chemical injury. CCL15/MIP-1δ has been shown to be expressed constitutively in the intestinal epithelial cells and play a role in chronic inflammatory pathologies of the intestine such as Crohn's disease and ulcerative colitis [54]. It is not clear whether its level could be elevated following surgery. This should be borne in mind if these markers are planned to be evaluated as biomarkers for either diagnosis or prognosis or follow-up.

In conclusion, our study has identified several genes differentially expressed in gastric cancers, some for the first time. Some of these have been confirmed at the protein level, as well. In addition, we have shown some of the proteins (CCL20/MIP-3α, TIMP1, IL8, PDGFR-B, CCL3/MIP-1α and CXCL9/MIG) to be detected at a higher level in the plasma from gastric cancer patients than in patients with non-malignant gastric conditions or with normal gastric mucosa. These will need to be evaluated further for their potential as diagnostic biomarkers in gastric cancers. A larger number of patients and a longer duration of follow-up will be required to obtain meaningful prognostic information particularly with regard to survival.

## Competing interests

The authors declare that they have no competing interests.

## Authors' contributions

TR conceived the study; acquired, analysed & interpreted the data and drafted the article. NV standardized and together with UMR performed the microarray experiments. GG standardized and together with UMR carried out the Quantibody array experiments. KS was involved in the acquisition and analysis of the microarray data. SS carried out all the pathological studies and assessment of samples for the microarray studies. SAR was involved in the clinical management and follow-up of the patients. All the authors read and approved the final version of the manuscript.

## Supplementary Material

Additional file 1 **Clinico-pathologic data of all the patients whose samples were used for microarray analysis**. The clinico-pathological details of all the patients (AN, PN and Cancers), whose samples were used for the microarray analysis are given. Click here for file

Additional file 2 **Clinico-pathologic data of only the AN and PN samples **.  The clinico-pathological details of only the AN and PN samples that were used for the microarray analysis are given. Click here for file

Additional file 3 **Clinico-pathologic details of the patients in whom the plasma levels of the cytokine/chemokine/growth factors were estimated**. The clinico-pathologic details of the patients from whom blood samples were obtained for estimation of the plasma levels of cytokines/chemokines/growth factors. Click here for file

Additional file 4 **Results of the Class Comparison analysis of the microarray data**. The file provides the results of the Class Comparison analysis done on the microarray data. Click here for file

Additional file 5 **Information on genes reported to be differentially expressed for the first time in gastric cancer** References and details of genes reported to be differentially expressed for the first time in gastric cancer. Click here for file

Additional file 6 **Information on genes known to be involved in gastric cancer tumorigenesis identified in this study**References and details of genes known to be involved in gastric cancer tumorigenesis identified in this study. Click here for file
