# Supplementary material for: Identification and validation of genes involved in gastric tumorigenesis
Source: Cancer Cell Int. 2010 Nov 24;10:45. doi: 10.1186/1475-2867-10-45 (PMC3004887; doi:10.1186/1475-2867-10-45)
Supplement: Additional file 5 — Information on genes reported to be differentially expressed for the first time in gastric cancer References and details of genes reported to be differentially expressed for the first time in gastric cancer. [file 1475-2867-10-45-S5.DOC]

| **TABLE 1: GENES IDENTIFIED FOR THE FIRST TIME AS BEING INVOLVED IN GASTRIC CANCER** | | | | |
| --- | --- | --- | --- | --- |
| **SNO** | **GENE SYMBOL** | **FUNCTION** | **UPREGULATION REPORTED IN** | **REFERENCE** |
| 1 | CCL15 / MIP5 / MIP-1d/ LKN1 | Chemotactic to monocytes and T cells. Binds to CCR1 and CCR3 | sEC, MMP-9, TIMP-1, and CCL15 levels correlate  with response to combination therapy with erlotinib and celecoxib in  patients with NSCLC | [SF5-R1] |
| 2 | ASPN | Can inhibit TGF-β and BMP2 protein | Upregulated in aromatase inhibitor treatment in breast cancer | [SF5-R2] |
| 3 | MMP3 | Breakdown of extracellular matrix | MMP1, MMP3 and MMP7 are overexpressed  in colorectal tumors | [SF5-R3] |
| 4 | SPON2 | Cell adhesion protein | Differentially expressed in cancerous and non- cancerous lung cells  Over expressed in ovarian cancer  Candidate marker of prostate cancer | [SF5-R4]  [SF5-R5]  [SF5-R6] |
| 5 | PRSS22 | Encodes a trypsinogen | Over-expressed in ovarian Cancer | [SF5-R7] |
| 6 | CCL3 / MIP 1A | A monokine, involved in the acute  inflammatory state in the recruitment and activation of polymorphonuclear leukocytes. | Overexpressed in oral squamous cell carcinoma | [SF5-R8] |
| 7 | TMEPAI / PMEPAI | Transmembrane prostate androgen-induced protein | Upregulated in Neuroendocrine tumours | [SF5-R9] |
| 8 | SIX3 | Involved in regulation of eye development |  |  |
| 9 | MFNG | Tumour suppressor in Notch pathway | Downregulated in cervical cancer | [SF5-R10] |
| 10 | SGNE1/SCG5 | Molecular chaperone | Overexpressed in endocrine tumors |  |
| 11 | SOSTDC1 | Modulates WNT and BMP signalling |  | [SF5-R11] |
| 12 | SST | Inhibits release of somatotrophin | Downregulated in oesophageal adenoca | [SF5-R12] |
| 13 | IGHA1 | Major immunoglobulin in body secretions |  |  |
| 14 | AKR1B10 | Can reduce aliphatic and aromatic aldehydes. Detoxification of reactive aldehydes | Increased in Barrett’s epithelium | [SF5-R13] |
| 15 | FCGBP | Maintenance of mucous structure | Down regulated in colon ca | [SF5-R14] |
| 16 | ATP4B | Proton pump beta chain |  |  |
| 17 | NCAPH2 | Regulatory subunit of condensing -2 complex |  |  |

**REFERENCES FOR TABLE 1**

1. Reckamp KL, Gardner BK, Figlin RA, Elashoff D, Krysan K, Dohadwala M, Mao J, Sharma S, Inge L, Rajasekaran A, Dubinett SM**.** Tumor response to combination celecoxib and erlotinib therapy in non-small cell lung cancer is associated with a low baseline matrix metalloproteinase-9 and a decline in serum-soluble E-cadherin. *J Thorac Oncol* 2008;**3:**117-24.

# [**Mackay A**](http://www.ncbi.nlm.nih.gov/pubmed?term="Mackay A"%5BAuthor%5D&itool=EntrezSystem2.PEntrez.Pubmed.Pubmed_ResultsPanel.Pubmed_RVAbstract), [**Urruticoechea A**](http://www.ncbi.nlm.nih.gov/pubmed?term="Urruticoechea A"%5BAuthor%5D&itool=EntrezSystem2.PEntrez.Pubmed.Pubmed_ResultsPanel.Pubmed_RVAbstract), [**Dixon JM**](http://www.ncbi.nlm.nih.gov/pubmed?term="Dixon JM"%5BAuthor%5D&itool=EntrezSystem2.PEntrez.Pubmed.Pubmed_ResultsPanel.Pubmed_RVAbstract), [**Dexter T**](http://www.ncbi.nlm.nih.gov/pubmed?term="Dexter T"%5BAuthor%5D&itool=EntrezSystem2.PEntrez.Pubmed.Pubmed_ResultsPanel.Pubmed_RVAbstract), [**Fenwick K**](http://www.ncbi.nlm.nih.gov/pubmed?term="Fenwick K"%5BAuthor%5D&itool=EntrezSystem2.PEntrez.Pubmed.Pubmed_ResultsPanel.Pubmed_RVAbstract), [**Ashworth A**](http://www.ncbi.nlm.nih.gov/pubmed?term="Ashworth A"%5BAuthor%5D&itool=EntrezSystem2.PEntrez.Pubmed.Pubmed_ResultsPanel.Pubmed_RVAbstract), [**Drury S**](http://www.ncbi.nlm.nih.gov/pubmed?term="Drury S"%5BAuthor%5D&itool=EntrezSystem2.PEntrez.Pubmed.Pubmed_ResultsPanel.Pubmed_RVAbstract), [**Larionov A**](http://www.ncbi.nlm.nih.gov/pubmed?term="Larionov A"%5BAuthor%5D&itool=EntrezSystem2.PEntrez.Pubmed.Pubmed_ResultsPanel.Pubmed_RVAbstract), [**Young O**](http://www.ncbi.nlm.nih.gov/pubmed?term="Young O"%5BAuthor%5D&itool=EntrezSystem2.PEntrez.Pubmed.Pubmed_ResultsPanel.Pubmed_RVAbstract), [**White S**](http://www.ncbi.nlm.nih.gov/pubmed?term="White S"%5BAuthor%5D&itool=EntrezSystem2.PEntrez.Pubmed.Pubmed_ResultsPanel.Pubmed_RVAbstract), [**Miller WR**](http://www.ncbi.nlm.nih.gov/pubmed?term="Miller WR"%5BAuthor%5D&itool=EntrezSystem2.PEntrez.Pubmed.Pubmed_ResultsPanel.Pubmed_RVAbstract), [**Evans DB**](http://www.ncbi.nlm.nih.gov/pubmed?term="Evans DB"%5BAuthor%5D&itool=EntrezSystem2.PEntrez.Pubmed.Pubmed_ResultsPanel.Pubmed_RVAbstract), et al. Molecular response to aromatase inhibitor treatment in primary breast cancer. *Breast Cancer Res* 2007;9:R37.

1. Lièvre A, Milet J, Carayol J, Le Corre D, Milan C, Pariente A, Nalet B, Lafon J, Faivre J, Bonithon-Kopp C, Olschwang S, Bonaiti-Pellié C, et al. Genetic polymorphisms of MMP1, MMP3 and MMP7 gene promoter and risk of colorectal adenoma**.** *BMC Cancer* 2006;**6**:270.
2. Manda R, Kohno T, Matsuno Y, Takenoshita S, Kuwano H, Yokota J. Identification of genes (SPON2 and C20orf2) differentially expressed between cancerous and noncancerous lung cells by mRNA differential display**.** *Genomics* 1999;**61**:5-14.

# Simon I, Liu Y, Krall KL, Urban N, Wolfert RL, Kim NW, McIntosh MW. Evaluation of the novel serum markers B7-H4, Spondin 2, and DcR3 for diagnosis and early detection of ovarian cancer. *Gynecol Oncol* 2007; 106:112-8.

1. Edwards S, Campbell C, Flohr P, Shipley J, Giddings I, te-Poele R, Dodson A, Foster C, Clark J, Jhavar S, Kovacs G, Cooper C S. Expression analysis onto microarrays of randomly selected cDNA clones highlights HOXB13 as a marker of human prostate cancer. *Br J Cancer* 2005; **92(2**): 376–381.
2. Paju A, Vartiainen J, Haglund C, Itkonen O, von Boguslawski K, Leminen A, Wahlström T, Stenman UH. Expression of trypsinogen-1, trypsinogen-2, and tumor-associated trypsin inhibitor in ovarian cancer: prognostic study on tissue and serum. [*Clin Cancer Res*](javascript:AL_get(this, 'jour', 'Clin Cancer Res.');) 2004;**10**:4761-8.
3. Silva TA, Ribeiro FL, Oliveira-Neto HH, Watanabe S, Alencar Rde C, Fukada SY, Cunha FQ, Leles CR, Mendonça EF, Batista AC. Dual role of CCL3/CCR1 in oral squamous cell carcinoma: implications in tumor metastasis and local host defense**.** *Oncol Rep* 2007;**18**:1107-13.
4. Hofsli E, Wheeler TE, Langaas M, Laegreid A, Thommesen L. Identification of novel neuroendocrine-specific tumour genes. *Br J Cancer* 2008;**99**:1330-9.
5. Veeraraghavalu K, Pett M, Kumar RV, Nair P, Rangarajan A, Stanley MA, Krishna S. Papillomavirus-mediated neoplastic progression is associated with reciprocal changes in JAGGED1 and manic fringe expression linked to notch activation. *J Virol* 2004;**78**:8687-700.
6. Blish KR, Wang W, Willingham MC, Du W, Birse CE, Krishnan SR, Brown JC, Hawkins GA, Garvin AJ, D'Agostino RB Jr, Torti FM, Torti SV. A human bone morphogenetic protein antagonist is down-regulated in renal cancer. *Mol Biol Cell* 2008;**19**:457-64.
7. Jin Z, Mori Y, Hamilton JP, Olaru A, Sato F, Yang J, Ito T, Kan T, Agarwal R, Meltzer SJ. Hypermethylation of the somatostatin promoter is a common, early event in human esophageal carcinogenesis**.***Cancer* 2008;**112**:43-49.
8. Breton J, Gage MC, Hay AW, Keen JN, Wild CP, Donnellan C, Findlay JB, Hardie LJ. Proteomic screening of a cell line model of esophageal carcinogenesis identifies cathepsin D and aldo-keto reductase 1C2 and 1B10 dysregulation in Barrett's esophagus and esophageal adenocarcinoma. *J Proteome Res* 2008;**7**:1953-62.
9. Lee S, Bang S, Song K, Lee I. Differential expression in normal-adenoma-carcinoma sequence suggests complex molecular carcinogenesis in colon**.** *Oncol Rep* 2006;**16**:747-54.
