# Supplementary material for: Identification and validation of genes involved in gastric tumorigenesis
Source: Cancer Cell Int. 2010 Nov 24;10:45. doi: 10.1186/1475-2867-10-45 (PMC3004887; doi:10.1186/1475-2867-10-45)
Supplement: Additional file 6 — Information on genes known to be involved in gastric cancer tumorigenesis identified in this studyReferences and details of genes known to be involved in gastric cancer tumorigenesis identified in this study. [file 1475-2867-10-45-S6.DOC]

TABLE 2: GENES KNOWN TO BE INVOLVED IN GASTRIC CANCER TUMORIGENESIS IDENTIFIED IN THIS STUDY

| **S NO** | **GENE SYMBOL** | **FUNCTION** | **UP OR DOWN REGULATED** | **REFERENCE** |
| --- | --- | --- | --- | --- |
| 1 | CTSB | Proteolytic enzyme involved in tumour invasion and metastasis. | Up-regulated especially in cardia tumours | [SF6-R1] |
| 2 | SPARC / osteonectin | Regulates cell growth through interactions with the extracellular matrix and  cytokines. Activates MMP2 at cell surface promoting invasion and metastasis | Up-regulated | [SF6-R2] |
| 3 | COL1A1 | Forms type 1 collagen | Up-regulated | [SF6-R3] |
| 4 | COL1A2 | Forms type 1 collagen | Up-regulated | [SF6-R3] |
| 5 | COL4A1 / Arresten | Forms type IV collagen chain of basement membranes | Up-regulated | [SF6-R4] |
| 6 | CXCL1 / GRO1 / MGSA | Has mitogenic properties and is implicated in melanoma pathogenesis; Involved in inflammatory response to H.Pylori | Up-regulated | [SF6-R5] |
| 7 | SPP1 / osteopontin | Acts as a cytokine involved in enhancing production of interferon-gamma and  interleukin-12 and reducing production of interleukin-10 and is essential in the pathway that leads to type I immunity | Up-regulated | [SF6-R6] |
| 8 | CXCL9 / MIG | Cytokine that affects the growth, movement, or activation state of cells that participate in immune and inflammatory response. Chemotactic for activated T-cells. Binds to CXCR3 | Up-regulated | [SF6-R7] |
| 9 | IL8 / CXCL8 | This gene is a member of the CXC chemokine family. This chemokine is one of the major mediators of the inflammatory response and a potent angiogenic factor | Up-regulated | [SF6-R8] |
| 10 | TIMP1 | Can inhibit MMP’s; has anti-apototic function; induce cell proliferation | Up-regulated | [SF6-R9] |
| 11 | LUM / SLRR2D / LDC | Regulates collagen fibril organization and circumferential growth, and epithelial cell migration and tissue repair. | Up-regulated | [SF6-R10] |
| 12 | CXCL5 / ENA78 | An inflammatory chemokine that belongs to the CXC chemokine family. This chemokine is produced concomitantly with interleukin-8 (IL8) in response to stimulation with either interleukin-1 (IL1) or tumour necrosis factor-alpha (TNF-A) | Up-regulated | [SF6-R11] |
| 13 | CXCL10 / INP10 / IP10 | Chemotactic for monocytes and T cells. Binds to CXCR3 | Up-regulated | [SF6-R4] |
| 14 | CEACAM6 | Cell adhesion molecule related to CEA | Up-regulated | [SF6-R12] |
| 15 | REGIV | Involved in gastric adenocarcinomas | Up-regulated | [SF6-R13] |
| 16 | S100A10 / ANX2L / CAL1L | Induces dimerization of ANXA2/p36 | Up-regulated | [SF6-R14] |
| 17 | SERPINH1 / HSP47 / CBP1 | Binds specifically to collagen and may be involved as a chaperone in collagen biosynthesis. Its expression is induced by heat shock. | Up-regulated | [SF6-R15] |
| 18 | CDH3 | Calcium-dependent cell-cell adhesion glycoprotein | Up-regulated | [SF6-R16 |
| 19 | TACSTD1 / EPCAM / CD326 | Calcium independent cell adhesion molecule | Up-regulated | [SF6-R17] |
| 20 | IFITM1 / LEU13 | Regulates cell growth | Up-regulated | [SF6-R18] |
| 21 | CTHRC1 | May act as a negative regulator of collagen matrix deposition | Up-regulated | [SF6-R19] |
| 22 | SULF1 | Exhibits arylsulfatase activity and highly specific endoglucosamine-6-sulfatase activity. It can remove sulfate from the C-6 position of glucosamine within specific subregions of intact heparin. | Up-regulated | [SF6-R20] |
| 23 | RNASE1 | Endonuclease | Down-regulated | [SF6-R21] |
| 24 | PGC | Hydrolyses a variety of protein | Down-regulated | [SF6-R22] |
| 25 | PGA5 | Proteolytic enzyme | Down-regulated | [SF6-R22] |
| 26 | GIF | Promotes absorption of vitamin B12 | Down-regulated | [SF6-R23] |
| 27 | LTF | Anti-inflammatory, inhibits cancer development and metastasis | Down-regulated | [SF6-R24] |
| 28 | TFF1 | Protects gastrointestinal mucosa from noxious agents | Down-regulated | [SF6-R25] |
| 29 | CLDN18 | Plays a major role in tight junction-specific obliteration of the intercellular space,  through calcium-independent cell-adhesion activity | Down-regulated | [SF6-R26] |
| 30 | CFD | Component of alternative complement pathway | Secreted by gastric ca cell lines | [SF6-R27] |
| 31 | GHRL / Obestatin | May be a ligand for GPR39. Reduces gastric emptying activity | Down-regulated | [SF6-R28] |
| 32 | LIPF | Gastric lipase | Down-regulated | [SF6-R29] |
| 33 | ANXA10 | Regulate cell growth and signal transduction | Down-regulated | [SF6-R30] |

**REFERENCES FOR TABLE 2**

1. Van Dekken H, Tilanus HW, Hop WC, Dinjens WN, Wink JC, Vissers KJ, Van Marion R. Array comparative genomic hybridization, expression array, and protein analysis of critical regions on chromosome arms 1q, 7q, and 8p in adenocarcinomas of the gastroesophageal junction. *Cancer Genet Cytogenet* 2009, **189**:37-42.
2. Wang CS, Lin KH, Chen SL, Chan YF, Hsueh S. Overexpression of SPARC gene in human gastric carcinoma and its clinic-pathologic significance. *Br J Cancer* 2004, **91**:1924-30.
3. Oue N, Hamai Y, Mitani Y, Matsumura S, Oshimo Y, Aung PP, Kuraoka K, Nakayama H, Yasui W. Gene expression profile of gastric carcinoma: identification of genes and tags potentially involved in invasion, metastasis, and carcinogenesis by serial analysis of gene expression. *Cancer Res* 2004, **64**:2397-405.
4. Jinawath N, Furukawa Y, Hasegawa S, Li M, Tsunoda T, Satoh S, Yamaguchi T, Imamura H, Inoue M, Shiozaki H, Nakamura Y. Comparison of gene-expression profiles between diffuse- and intestinal-type gastric cancers using a genome-wide cDNA microarray. *Oncogene* 2004, **23**:6830-44.
5. Eck M, Schmausser B, Scheller K, Brändlein S, Müller-Hermelink HK. Pleiotropic effects of CXC chemokines in gastric carcinoma: differences in CXCL8 and CXCL1 expression between diffuse and intestinal types of gastric carcinoma. [*Clin Exp Immunol*](javascript:AL_get(this, 'jour', 'Clin Exp Immunol.');) 2003, **134**:508-15.
6. Zhang DT, Yuan J, Yang L, Guo XN, Hao ZM, Han ZY, Wu KC, Fan DM. [Osteopontin expression and its relation to invasion and metastases in gastric cancer](http://www.ncbi.nlm.nih.gov/pubmed/15946568). *Zhonghua Zhong Liu Za Zhi* 2005, **27**:167-9.
7. [Ohtani H](http://www.ncbi.nlm.nih.gov/pubmed?term="Ohtani H"%5BAuthor%5D&itool=EntrezSystem2.PEntrez.Pubmed.Pubmed_ResultsPanel.Pubmed_RVAbstract), Jin Z, Takegawa S, Nakayama T, Yoshie O. Abundant expression of CXCL9 (MIG) by stromal cells that include dendritic cells and accumulation of CXCR3+ T cells in lymphocyte-rich gastric carcinoma. *J Pathol* 2009, **217**:21-31.
8. Kitadai Y, Takahashi Y, Haruma K, Naka K, Sumii K, Yokozaki H, Yasui W, Mukaida N, [Ohmoto Y](http://www.ncbi.nlm.nih.gov/pubmed?term="Ohmoto Y"%5BAuthor%5D&itool=EntrezSystem2.PEntrez.Pubmed.Pubmed_ResultsPanel.Pubmed_RVAbstract), Kajiyama G, Fidler IJ, Tahara E. Transfection of interleukin-8 increases angiogenesis and tumorigenesis of human gastric carcinoma cells in nude mice**.** [*Br J Cancer*](javascript:AL_get(this, 'jour', 'Br J Cancer.');) 1999, **81**:647-53.
9. Mroczko B, Lukaszewicz-Zajac M, Groblewska M, Czyzewska J, Gryko M, Guzińska-Ustymowicz K, Kemona A, Kedra B, Szmitkowski M. Expression of tissue inhibitors of metalloproteinase 1 (TIMP-1) in gastric cancer tissue. Folia Histochem Cytobiol. 2009 Jan;47(3):511-6.
10. Takeno A, Takemasa I, Doki Y, Yamasaki M, Miyata H, Takiguchi S, Fujiwara Y, Matsubara K, Monden M. Integrative approach for differentially overexpressed genes in gastric cancer by combining large-scale gene expression profiling and network analysis. [*Br J Cancer*](javascript:AL_get(this, 'jour', 'Br J Cancer.');) 2008, **99**:1307-15.
11. Park JY, Park KH, Bang S, Kim MH, Lee JE, Gang J, [Koh SS](http://www.ncbi.nlm.nih.gov/pubmed?term="Koh SS"%5BAuthor%5D&itool=EntrezSystem2.PEntrez.Pubmed.Pubmed_ResultsPanel.Pubmed_RVAbstract), Song SY. CXCL5 overexpression is associated with late stage gastric cancer. [*J Cancer Res Clin Oncol*](javascript:AL_get(this, 'jour', 'J Cancer Res Clin Oncol.');) 2007, **133**:835-40.
12. Yasui W, Oue N, Ito R, Kuraoka K, Nakayama H. Search for new biomarkers of gastric cancer through serial analysis of gene expression and its clinical implications. *Cancer Sci* 2004, 95:385-92.
13. Oue N, Aung PP, Mitani Y, Kuniyasu H, Nakayama H, Yasui W. Genes involved in invasion and metastasis of gastric cancer identified by array-based hybridization and serial analysis of gene expression. *Oncology*  2005, **69**:17-22.
14. El-Rifai W, [Moskaluk CA](http://www.ncbi.nlm.nih.gov/pubmed?term="Moskaluk CA"%5BAuthor%5D&itool=EntrezSystem2.PEntrez.Pubmed.Pubmed_ResultsPanel.Pubmed_RVAbstract), [Abdrabbo MK](http://www.ncbi.nlm.nih.gov/pubmed?term="Abdrabbo MK"%5BAuthor%5D&itool=EntrezSystem2.PEntrez.Pubmed.Pubmed_ResultsPanel.Pubmed_RVAbstract), Harper J, Yoshida C, Riggins GJ, Frierson HF Jr, [Powell SM](http://www.ncbi.nlm.nih.gov/pubmed?term="Powell SM"%5BAuthor%5D&itool=EntrezSystem2.PEntrez.Pubmed.Pubmed_ResultsPanel.Pubmed_RVAbstract). Gastric cancers overexpress S100A calcium-binding proteins. *Cancer Res* 2002, **62**:6823-6.
15. Hirai K, Kikuchi S, Kurita A, Ohashi S, Adachi E, Matsuoka Y, Nagata K, Watanabe M. Immunohistochemical distribution of heat shock protein 47 (HSP47) in scirrhous carcinoma of the stomach. [*Anticancer Res*](javascript:AL_get(this, 'jour', 'Anticancer Res.');) 2006, **26**:71-8.
16. Imai K, Hirata S, Irie A, Senju S, Ikuta Y, Yokomine K, Harao M, Inoue M, Tsunoda T, Nakatsuru S, Nakagawa H, Nakamura Y, Baba H, Nishimura Y. Identification of a novel tumor-associated antigen, cadherin 3/P-cadherin, as a possible target for immunotherapy of pancreatic, gastric, and colorectal cancers. [*Clin Cancer Res*](javascript:AL_get(this, 'jour', 'Clin Cancer Res.');) 2008, **14**:6487-95.
17. Wenqi D, Li W, Shanshan C, Bei C, Yafei Z, Feihu B, Jie L, Daiming F. EpCAM is overexpressed in gastric cancer and its downregulation suppresses proliferation of gastric cancer. *J Cancer Res Clin Oncol* 2009, **135**:1277-85.
18. Yang Y, Lee JH, Kim KY, Song HK, Kim JK, Yoon SR, Cho D, Song KS, Lee YH, Choi I. The interferon-inducible 9-27 gene modulates the susceptibility to natural killer cells and the invasiveness of gastric cancer cells. *Cancer Lett* 2005, **221**:191-200.
19. Tang L, Dai DL, [Su M](http://www.ncbi.nlm.nih.gov/pubmed?term="Su M"%5BAuthor%5D&itool=EntrezSystem2.PEntrez.Pubmed.Pubmed_ResultsPanel.Pubmed_RVAbstract), Martinka M, Li G, Zhou Y. Aberrant expression of collagen triple helix repeat containing 1 in human solid cancers*.* [*Clin Cancer Res*](javascript:AL_get(this, 'jour', 'Clin Cancer Res.');) 2006, **12**:3716-22.
20. Junnila S, Kokkola A, Mizuguchi T, Hirata K, Karjalainen-Lindsberg ML, Puolakkainen P, Monni O. Gene expression analysis identifies over-expression of CXCL1, SPARC, SPP1, and SULF1 in gastric cancer. *Genes Chromosomes Cancer* 2010, **49**:28-39.
21. Wang L, Zhu JS, Song MQ, Chen GQ, Chen JL. Comparison of gene expression profiles between primary tumor and metastatic lesions in gastric cancer patients using laser microdissection and cDNA microarray. *World J Gastroenterol* 2006, **12**:6949-54.
22. Ning PF, Sun LP, Liu HJ, Yuan Y. Expression of pepsinogen C in gastric cancer and its precursor. *Zhonghua Yi Xue Za Zhi* 2004, **84**:818-21.
23. El-Rifai W, Frierson HF Jr, Harper JC, Powell SM, Knuutila S. Expression profiling of gastric adenocarcinoma using cDNA array. *Int J Cancer* 2001, **92**:832-8.
24. Luqmani YA, Campbell TA, Bennett C, Coombes RC, Paterson IM. Expression of lactoferrin in human stomach. *Int J Cancer* 1991, **49**:684-7.

# Carvalho R, Kayademir T, Soares P, Canedo P, Sousa S, Oliveira C, Leistenschneider P, Seruca R, Gött P, Blin N, Carneiro F, Machado JC. Loss of heterozygosity and promoter methylation, but not mutation, may underlie loss of TFF1 in gastric carcinoma. *Lab Invest* 2002, 82:1319-26.

1. Sanada Y, Oue N, Mitani Y, Yoshida K, Nakayama H, Yasui W. Down-regulation of the claudin-18 gene, identified through serial analysis of gene expression data analysis, in gastric cancer with an intestinal phenotype. [*J Pathol*](javascript:AL_get(this, 'jour', 'J Pathol.');) 2006, **208**:633-42.
2. Kitano E, Kitamura H. Synthesis of factor D by gastric cancer-derived cell lines. *Int Immunopharmacol* 2002;**2**:843-8.
3. An JY, Choi MG, Noh JH, Sohn TS, Jin DK, Kim S. Clinical significance of ghrelin concentration of plasma and tumor tissue in patients with gastric cancer. *J Surg Res.* 2007, **143**:344-9.
4. Sun XJ, Sun KL, Zheng ZH, Fu WN, Hao DM, Xu HM, [Li XM](http://www.ncbi.nlm.nih.gov/pubmed?term="Li XM"%5BAuthor%5D&itool=EntrezSystem2.PEntrez.Pubmed.Pubmed_ResultsPanel.Pubmed_RVAbstract). Gene expression patterns in gastric cancer. [*Zhonghua Yi Xue Yi Chuan Xue Za Zhi*](javascript:AL_get(this, 'jour', 'Zhonghua Yi Xue Yi Chuan Xue Za Zhi.');) 2006, **23**:142-6.
5. Kim J, Kim MA, [Jee CD](http://www.ncbi.nlm.nih.gov/pubmed?term="Jee CD"%5BAuthor%5D&itool=EntrezSystem2.PEntrez.Pubmed.Pubmed_ResultsPanel.Pubmed_RVAbstract), Jung EJ, Kim WH. Reduced expression and homozygous deletion of annexin A10 in gastric carcinoma*. Int J Cancer* 2009, **125**:1842-50.
